# Vaccines against RNA Viruses

**DOI:** 10.3390/vaccines8030479

**Published:** 2020-08-27

**Authors:** Juan-Carlos Saiz

**Affiliations:** ZOOVIR, Department of Biotechnology, Instituto Nacional de Investigaciones Agrarias (INIA), 28040 Madrid, Spain; jcsaiz2012@gmail.com

RNA viruses cause animal, human, and zoonotic diseases that affect millions of individuals, as is being exemplified by the devastating ongoing epidemic of the recently identified SARS-Cov-2. For years vaccines have had an enormous impact on overcoming the global burden of diseases. Nowadays, a vast number of different approaches, from purified inactivated and live attenuated viruses, nucleic acid (DNA or RNA) based candidates, virus-like particles, subunit elements, and recombinant viruses are been employed to combat diverse diseases caused by RNA viruses. However, although for some of them efficient vaccines are available, in most instances, and for different reasons (technologic o economic restrictions, etc.), they are scarcely used in the field, and even for many of them no licensed vaccines exist. This will probably change dramatically with the current Covid-19 pandemic, as a vast variety of vaccinology approaches are being tested against it, with hundreds of candidates under development, dozens of them already in clinical trials phase III and IV, a fact that is breaking records in vaccine development and implementation. This is becoming possible thanks to the enormous work carried out during years to have the bases for a quick response, even against unknown pathogens, in an impressive short time.

In this *Vaccines* Special Issue “Vaccines against RNA viruses”, results obtained with different vaccine methodological approaches against human, animal, and zoonotic viruses are presented by field experts.

Human Immunodeficiency Virus (HIV), for which no vaccine is available, continues to be a major global public health issue that has already cost almost 33 million lives so far, and with an estimated 38 million people living with it. Even though antiretroviral therapy considerably prolongs the lifespan of patients making the disease a manageable chronic health condition, treatment is expensive and is not available for all infected patients. Thus, vaccines are needed to control HIV spread. So, in this Issue, Rudometov et al. [1] describe the development of a human immunodeficiency virus-1 (HIV-1) immunogen using a polypeptide recombinant non transferrin bound iron (nTBI) protein carrying four T- and five B-cell epitopes from HIV-1 Env and Gag proteins recognized by broadly neutralizing HIV-1 antibodies, combined with Th-epitopes. Immunization of rabbits with this nTBI protein elicited antibodies that recognize HIV-1 proteins, supporting its possible use as immunogen.

Another relevant human virus is the human rotavirus (HRV) that is a leading cause of severe, dehydrating gastroenteritis, mainly in children. Current live rotavirus vaccines are efficient, but costly, and they may present increased risk of intussusception due to vaccine replication in the gut of vaccinated children. Here, Ramesh and coworkers [2] assess the immunogenicity and protective capacity of a novel P24-VP8* nanoparticle vaccine using the gnotobiotic (Gn) pig model of human rotavirus. Three doses (200 μg/dose) of the vaccine candidate intramuscularly administered with Al(OH)_3_ (600 μg) as an adjuvant conferred significant protection against infection and diarrhea after challenged with virulent heterologous rotavirus strains. Vaccinated animals showed a significant reduction in the mean duration of diarrhea, virus shedding in feces, and significantly lower fecal cumulative consistency scores in comparison with mock immunized control animals. Vaccinated animals also elicited strong VP8*-specific serum neutralizing antibody responses, but, consistent with the methodological approach (immunization route, adjuvant, and lack of replication), no serum or intestinal IgA antibody responses, or strong effector T cell responses, were induced. Authors indicate that their results open the option of the initiation of clinical trials using the new designed P24-VP8* nanoparticle vaccine candidate.

For his part, Duncan and coworkers [3] update the current status of the hepatitis C vaccination (HCV), describing and discussing the different ongoing research in the field, and emphasizing that, despite the great advances in HCV therapeutics achieved lately, treatments sometime fail to prevent reinfection and are quite expensive and, thus, efficient prophylactic HCV vaccines are still needed.

Pigs are one of the main livestock species, comprising 36% of the worldwide meat consumption, with hundreds of tons produced yearly. Pigs can be infected by a wide variety of RNA viruses that cause huge losses for the food industry. In addition, they can serve as bridge between wild host animals and humans. Thereby, availability of efficient vaccines is crucial. Among the viral infections affecting pigs, a very important one is that of the porcine reproductive and respiratory syndrome virus (PRRSV) that causes reproductive failure in pregnant sows and respiratory disease in young pigs, accounting for huge economic losses to industry across the world. One critical drawback for vaccine development against PRRSV is its high genetic diversity. Here, Ciu and coworkers [4] show their results after testing a glycoprotein GP5-Mosaic DNA vaccine and a recombinant GP5-Mosaic vaccinia virus (rGP5-Mosaic VACV) vaccine in pigs, and compare their immunogenicity and protective capacity against heterologous virulent strains (VR2332 or MN184C) with that of a rGP5-wyld-type, WT (VR2332) DNA and a rGP5-WT (VR2332) VACV vaccine, using as controls groups those inoculated with empty vector DNA or empty VACV. The authors show that vaccination with the GP5-Mosaic-based vaccines resulted in cellular reactivity and higher levels of neutralizing antibodies to both VR2332 and MN184C PRRSV strains, whilst vaccination with the GP5-WT vaccines only induced response against the heterologous challenging virus (VR2332). After infection with either strain, viral titers in sera and tissues, as well as lung lesions, were lower in the GP5-Mosaic vaccinated pigs. These results indicate that, using a DNA-prime/VACV boost regimen, the developed GP5-Mosaic candidates confer protection in pigs against heterologous viruses and, thus, are feasible vaccine candidates.

Another important disease in pigs is that caused by the porcine epidemic diarrhea virus (PEDV), a coronavirus responsible of highly contagious intestinal infections that may result in the death of newborn piglets and weight loss in pigs of all ages, and that seriously damages the swine industry. From 2010, novel highly virulent PEDV genotype 2 variants have spread through China, resulting in high mortality of newborn piglets and huge economic losses. Current vaccines against the classical CV777 strain of genotype 1 do not provide effective protection against Chinese highly virulent PEDV variant infections. Thus, Shi and colleagues [5] using a RNA interference (RNAi) approach, show that three short hairpin RNA (shRNA)-expressing plasmids directed against the viral nucleocapsid (N) present antiviral activities in intestine epithelial cells infected with a both classical CV777 and LNCT2 strains, and that these shRNAs markedly reduce viral replication of both of strains upon downregulation of N protein production; thus, these strategies, based on targeting viral processivity factors, may be feasible vaccine alternatives.

By a different approach, Cañas-Arranz et al. [6] deepen their many years of previous research with peptides as vaccine candidates against foot-and mouth disease virus (FMDV). The virus is responsible for a highly contagious transmissible infection of cloven-hoofed animals (mainly pigs and cows) that may cause huge economic impact and whose control relies on efficient vaccination using a conventional chemically inactivated virus. However, these current vaccines present limitations, as the need for a strict maintenance of the cold chain and for a constant update of vaccine strains due to the virus’s antigenic diversity, as well as the fact that the manufacturing process poses significant biosafety concerns, as it has been related to occasional escape episodes. Therefore, vaccines incorporating specific outbreak-relevant epitopes capable of eliciting protective and quick responses can become an invaluable emergency resource for FMD containments during outbreaks. With this background, the Spanish group go one step further by testing in pigs a dendrimer peptide B2T with two copies of a B-cell VP1 epitope linked through maleimide units to a T-cell 3A, and show that a single dose of B2T evokes a specific protective immune response with high neutralizing titers, activates the T cell response, induces IFN production, and fully protects 70% of vaccinated pigs that did not present clinical signs of the disease. Their results strengthen the potential of B2T as a safe, cost-effective candidate vaccine, which can be of particular interest in emergency scenarios.

Rift Valley fever virus (RVFV), a mosquito-borne bunyavirus widely distributed in Sub-Saharan countries, Egypt, and the Arabian Peninsula, is another important pathogen causing disease in humans, in which severe cases can end in encephalitis or hemorrhagic fever, and in ruminant livestock, characterized by an increased incidence of abortion or fetal malformation. At present, there are a few veterinary vaccines available for use in endemic areas, but there is no licensed human vaccine. López-Gil and coworkers [7], by using an approach based on the modified vaccinia Ankara (MVA) encoding the RVFV glycoproteins (rMVAGnGc), extend their previous observations that a single inoculation was sufficient to induce a protective immune response in mice after a lethal viral challenge, which was related to the presence of glycoprotein specific CD8+ cells and a low-level detection of in vitro neutralizing antibodies. They tested the efficacy and immune response in mice immunized with recombinant MVA viruses expressing either glycoprotein Gn (rMVAGn) or Gc (rMVAGc) and suggest that, in the absence of serum neutralizing antibodies, protection is strain-dependent and mainly due to the activation of the cellular response against Gc epitopes. Even more, their data point to the induction of a suboptimal humoral immune response, since disease was exacerbated upon the virus challenge in the presence of rMVAGnGc or rMVAGn immune serum. These results support that Gc-specific cellular immunity is an important component that contributes to efficient protection against RVFV infection.

The fishing industry is increasingly relevant, and the number of farms has grown exponentially, being a very important source of food. Viral hemorrhagic septicemia virus (VHSV), a novirhabdovirus, is one of the worst viral threats to fish farming. VHSV has been isolated from more than 50 fish species across the world, including farmed and free-living marine species. Detection of even a single positive sample in a farm has to be notified to the Office International des Epizooties, and implies the sacrifice of all the farmed fish, thus leading to serious economic losses. Non-virion (NV) gene-deleted VHSV (dNV-VHSV) has been postulated as an attenuated virus, as its absence leads to lower induced pathogenicity. In the study by Chinchilla et al. [8] the immune transcriptome profiling in trout infected with dNV-VHSV and wt-VHSV and the pathways involved in immune responses were analyzed in the context of infection. The authors show that dNV-VHSV upregulates more trout-signaling immune related genes and pathways, whereas wt-VHSV maintains more non-regulated genes. Therefore, wt-VHSV impairs the activation at short stages of infection of pro-inflammatory, antiviral, proliferation, and apoptosis pathways, delaying innate humoral response and cellular crosstalk, whereas dNV-VHSV promotes the opposite effects, supporting the use of dNV-VHSV as a potential live vaccine candidate.

Finally, Jiménez de Oya and coworkers [9] deeply update current knowledge and available data about the vaccination of birds against the West Nile virus (WNV), the worldwide most distributed mosquito-borne flavivirus. Although humans and equids can sporadically be infected, birds are the natural host of WNV. When clinical signs arise in birds it is due to multi-organ invasion, mainly in the central nervous system, which can lead to death 24–48 h later. Nowadays, vaccines are only available for use in equids; thus, availability of avian vaccines would benefit bird populations, both domestic and wild ones. Such vaccines could be used in endangered species housed in rehabilitation and wildlife reserves, and in animals located at zoos and other recreational installations, but also in farm birds, and in those that are grown for hunting and restocking activities. Even more, controlling WNV infection in birds can also be useful to prevent its spread and limit outbreaks. In their review, Jimenez de Oya and colleagues comprehensively present the results obtained with commercial and experimental vaccines in domestic and wild avian species, and the possible benefits and drawbacks of bird vaccination against WNV are discussed.

The world remains burdened by high morbidity and mortality diseases and, as exemplified by the current devastating pandemic of SARS-Cov-2, new emerging or re-emerging pathogens are likely to spread in the future. Hereby, a more comprehensive understanding of the current trends in vaccine development and assessment of the molecular mechanisms and immune responses involved in the elicited responses are essential. In this line, the articles in this Issue highlight recent advances in the development of efficient vaccines against RNA viruses infecting animals and humans, some of which are zoonotic. Different approaches are described from attenuated and recombinant viruses, to peptides, and DNA and RNA-based candidates, which hopefully will contribute to a better and quick preparedness against RNA virus infections.
